# Exploring the mechanism of proteinuria reduction by hydroxychloroquine in IgA nephropathy using network pharmacology and molecular mocking

**DOI:** 10.1038/s41598-025-97950-z

**Published:** 2025-07-01

**Authors:** Meiqi Lu, Yuxin Wang, Zheng Wan, Yixuan Chen, Mengshu Lin, Xiaoqi Deng, Xiaoxia Su, Qing Gao

**Affiliations:** 1 Department of Nephrology, Zhongshan Hospital of Xiamen University, School of Medicine, Xiamen University, Xiamen, China; 2 Department of Nephrology, Xiang’an Hospital of Xiamen University, School of Medicine, Xiamen University, Xiamen, China; 3 Department of Nephrology, Zigong Fourth People’s Hospital, Zigong, Sichuan Province China

**Keywords:** Network pharmacology, IgA nephropathy, Hydroxychloroquine, miR-130b-3p, Biomarkers, Drug regulation, Diseases, Medical research, Nephrology

## Abstract

According to the 2021 KDIGO guidelines, hydroxychloroquine (HCQ) had been recommended for the treatment of IgA nephropathy (IgAN). However, the precise mechanisms by which HCQ ameliorated proteinuria in IgAN were not fully understood. This study investigated the potential mechanisms of HCQ in reducing proteinuria in IgAN using network pharmacology and molecular docking approaches. Targets associated with IgAN, proteinuria, and HCQ were identified from databases including DisGeNET, GeneCards, OMIM, and PharmMapper. GO and KEGG analyses were conducted using the DAVID platform. Protein–protein interaction (PPI) networks were constructed using the STRING database, and hub genes were identified using Cytoscape software. The selection of hub genes was corroborated with data from the GEO database and validated through molecular docking. Additionally, miRNAs were predicted using NetworkAnalyst. A total of 48 genes were identified as being associated with the reduction of proteinuria in IgAN. The findings suggested that HCQ’s mechanism of action in mitigating proteinuria in IgAN primarily involved pathways related to inflammation. Furthermore, this mechanism was linked to the regulatory effects of miR-130b-3p on the expression of genes such as MMP2, IGF1, and PPARG. HCQ targeted miR-130b-3p, thereby influencing the TLR/MyD88/NF-κB signaling pathway and modulating the expression of MMP2, IGF1, and PPARG. This action may have been responsible for the observed reduction in proteinuria associated with IgAN.

## Introduction

IgA nephropathy (IgAN) is a common form of primary glomerulonephritis worldwide and a major cause of renal failure^1^. Proteinuria is a risk factor for impaired renal function in nephropathies and is associated with poor prognostic outcomes in IgAN^2^. Short-term reduction of proteinuria has been shown to significantly decrease the risk of disease progression in IgAN. In clinical practice, proteinuria reduction is often used as a surrogate outcome measure to evaluate the efficacy of IgAN treatments^3^. Angiotensin-converting enzyme inhibitors (ACEIs) and angiotensin receptor blockers (ARBs) are the mainstay of therapy for reducing proteinuria in IgAN. However, despite standard treatment with ACEIs/ARBs, more than 50% of patients still present with proteinuria levels exceeding 1 g/d^4^. Even after treatment with glucocorticoids, immunosuppressants, and biologics, proteinuria in some patients remains unimproved^5^. Therefore, it is essential to explore additional treatment strategies that can effectively and safely reduce proteinuria in IgAN patients.

Hydroxychloroquine (HCQ), a well-established antimalarial drug, has gained widespread application in the treatment of autoimmune diseases, exerting its effects through anti-inflammatory and immunomodulatory mechanisms^6^. Recent studies have demonstrated that HCQ could significantly reduce proteinuria in patients with IgAN^7–9^. Although its efficacy in reducing proteinuria might not have been as potent as that of glucocorticoids, immunosuppressants, and biologics, its favorable safety profile, characterized by fewer adverse reactions, had garnered ongoing interest in the treatment of IgAN^10^. According to the 2021 KDIGO guidelines, HCQ is recommended for Chinese IgAN patients at high risk of progressing to chronic kidney disease (CKD), even with adequate supportive management^11^. However, the specific mechanism by which HCQ reduces proteinuria in IgAN remains unclear. However, the specific mechanism by which HCQ reduced proteinuria in IgAN had remained unclear.

**Fig. 1:**
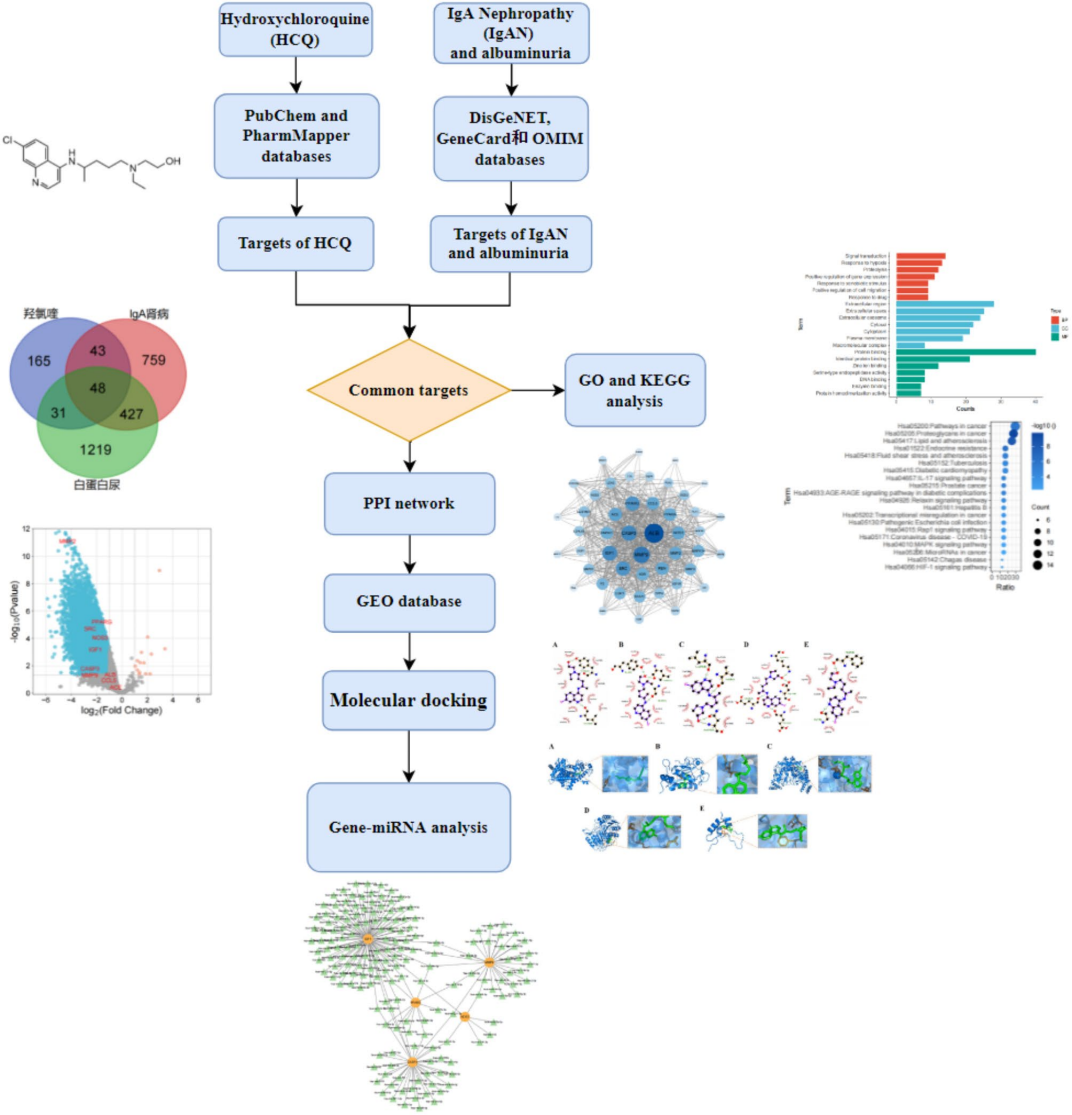
A flowchart illustrating the process of HCQ alleviating proteinuria in IgAN.

MicroRNA (miRNA) is identified as a key factor in the gene expression regulatory network. It regulate the expression of multiple genes, thereby influencing cell function and the occurrence and development of diseases^12^. The expression level of miRNA can be detected in blood and urine, which exhibited high stability and resistance to degradation. Consequently, miRNA is considered a potential biomarker for disease and a candidate for drug targeting^13^. MiRNA plays a significant regulatory role in the pathogenesis, progression, diagnosis, and management of IgAN^14^. Network pharmacology (NP), an integrative analytical approach bases on the interplay of disease, gene, drug, and target protein interaction networks, is employed to elucidate the mechanisms of drug action by integrating systems biology and computational biology with drug target-related “omics” data^15^. Several studies had applied NP to dissect the mechanisms of drug action in renal diseases, including IgAN and diabetic nephropathy (DN)^16,17^. Therefore, to uncover the molecular mechanisms by which HCQ reduced proteinuria in IgAN and to lay a theoretical groundwork for subsequent investigations, a NP analysis was performed in this study (Fig. 1).

## Materials and methods

### IgAN and albuminuria targets prediction

The DisGeNET^18^, GeneCard^19^, and Online Mendelian Inheritance in Man (OMIM)^20^ are powerful databases of human genes. With “IgA nephropathy” and “albuminuria” as keywords, the DisGeNET, GeneCard, and OMIM databases were searched for disease-related genes.

### HCQ targets prediction and common targets intersection

**Fig. 2:**
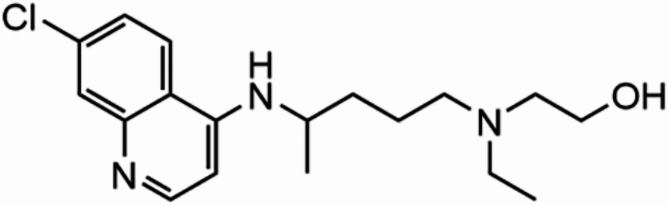
Chemical structure of HCQ.

The PubChem database contains data on over 100 million chemical substances, encompassing various types such as organic small molecules and bioactive molecules^21^. PharmMapper database is a highly valuable bioinformatics tool that focuses on serving the fields of drug development and molecular mechanism exploration. It relies on a massive built-in pharmacophore model to quickly and accurately predict potential drug target proteins that small molecules may act on^22^. UniProt, with its vast and authoritative protein database resources, collectes and organizes standard gene name information across a wide range of species, as well as corresponding detailed gene annotations^23^. The 2D and 3D chemical structures of HCQ were downloaded from the PubChem database, and the PharmMapper database was utilized for uploading the data (Fig. 2). The maximum conformation generation number in PharmMapper was set to 300. UniProt was employed to convert target gene identifiers into official names. A Venn diagram was constructed to identify the shared target genes between diseases and drugs^24^.

### GO and KEGG pathway analyses

The DAVID database is a powerful assistant in the field of bioinformatics, focusing on gene and protein information processing, including gene function annotation and gene pathway enrichment analysis^25^. To determine the key pathways influencing the reduction of albuminuria in IgAN by HCQ, the DAVID database was utilized for GO and KEGG analyses^26^. The results with *P* < 0.05 were evaluated using the Hiplot online platform and subsequently used for further analyses^27^.

### Construction of a PPI network

The STRING database is a protein interaction network database widely used in the field of bioinformatics, encompassing a broad spectrum of data sources that includes experimentally validated interactions, computationally predicted interactions, literature mining, and information from other databases^28^. The targets were submitted to the STRING database to construct a protein–protein interaction (PPI) network. Data visualization and network analysis were conducted using Cytoscape 3.9.1 software, followed by the use of the cytoHubba plugin to identify core proteins.

### Verification of the GEO dataset

The GEO database is a public database established and maintained by the National Center for Biotechnology Information (NCBI), playing a significant role in bioinformatics research. The database contains a wide range of data sources, including high-throughput gene expression data submitted by research institutions globally^29^. The dataset of human samples was searched using the keyword “IgA nephropathy” in the GEO database. Differential expression analysis was conducted using the GEO2R online tool, with the application of screening criteria “*P* < 0.05” and “|log2FC| > 2” to identify differentially expressed genes (DEGs). Subsequent to this analysis, a volcano plot was generated for annotating the core genes.

### Molecular docking

Molecular docking (MD) is a computational technique used to predict interaction and binding patterns between molecules, with the aim of studying and optimizing the binding modes and mechanical interactions between compounds and proteins^30^. Autodocktools 1.5.7 was utilized to assess the interactions between the key targets (receptors) and HCQ (ligand), and PyMOL was employed for visualization purposes. The HCQ structure was extracted from the PubChem database, while the target structure was sourced from the Protein Data Bank (PDB)^31^. A Lamarckian genetic algorithm search was performed to execute 50 runs. A binding energy less than − 5 kcal/mol was indicative of good docking activity^32^, and proteins that stably bound to HCQ were selected as potential targets for their role in mitigating albuminuria in IgAN.

### Construction of MiRNA networks

NetworkAnalyst integrates a substantial body of experimentally validated or computationally predicted miRNA-gene interaction data and employes corresponding algorithms to analyze input gene and miRNA datasets, thereby identifying potential interaction relationships^33^. In an effort to further elucidate the biological markers and miRNA regulatory mechanisms associated with HCQ’s role in reducing proteinuria in IgAN, genes that stably docked with HCQ were imported into the NetworkAnalyst database for gene-miRNA network analysis (Table 1). The prediction of miRNAs through genes is founded on sequence complementarity, which involves forecasting interactions between miRNAs and their target gene mRNAs based on this complementarity. Such complementary pairing may result in mRNA degradation or translation inhibition, thus modulating gene expression. Numerous studies had previously employed gene-miRNA network analysis to forecast biological markers and target miRNAs for conditions such as polycystic ovary syndrome^34^, kidney stones^35^, systemic lupus erythematosus^36^, and Parkinson’s disease^37^. Visualization and comprehensive analysis of the gene-miRNA network were performed using Cytoscape 3.9.1 software to identify candidate miRNAs.

**Table 1:** List of databases with corresponding urls.

Number	Database	Website
1	DisGeNET	https://www.disgenet.org/
2	GeneCard	https://www.genecards.org/
3	OMIM	https://omim.org/
4	PubChem	http://pubchem.ncbi.nlm.nih.gov/
5	PharmMapper	http://lilab-ecust.cn/pharmmapper/index.html/
6	UniProt	https://www.uniprot.org/
7	Venn	http://bioinformatics.psb.ugent.be/webtools/Venn/
8	STRING	https://string-db.org/
9	DAVID	https://david.ncifcrf.Gov/
10	Hiplot	https://hiplot-academic.com/
11	NetworkAnalyst	https://www.networkanalyst.ca/
12	GE02R	https://www.ncbi.nlm.nih.gov/geo/geo2r/
13	PDB	https://www.rcsb.org/pages/contactusPDB

## Results

### Preparation of the target

**Fig. 3:**
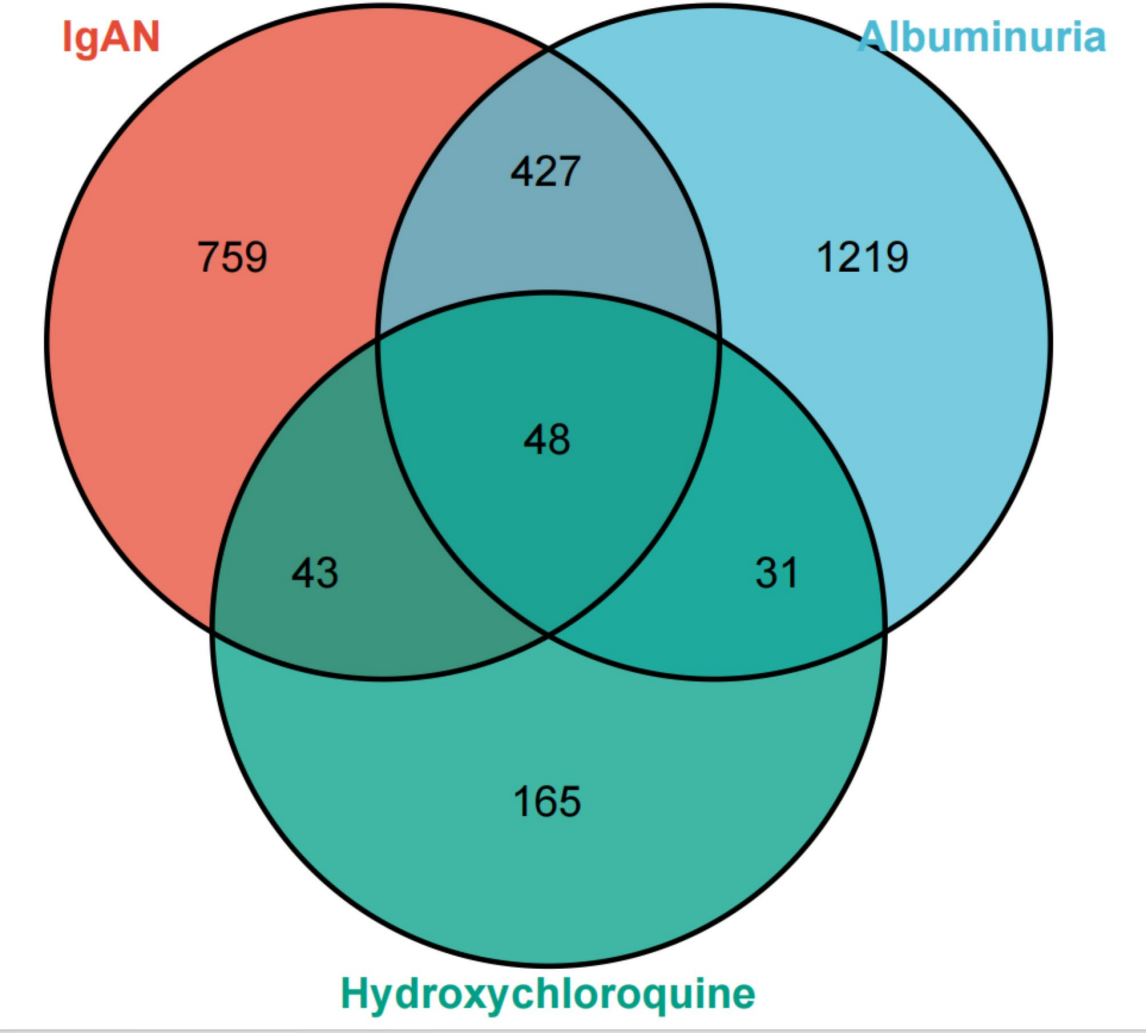
Venn diagram depicting overlapping genes associated with HCQ, IgAN, and Albuminuria”.

After duplicates were eliminated, 1,277 and 268 targets associated with IgAN and albuminuria were obtained from the OMIM, DisGeNET, and GeneCards databases, respectively. Following confirmation of the results with the UniProt database, 287 HCQ targets were identified based on the PharmMapper database. Diseases and drug targets were ascertained using the Venn platform, which disclosed 48 common targets (Fig. 3).

### Enrichment of GO and KEGG

**Fig. 4:**
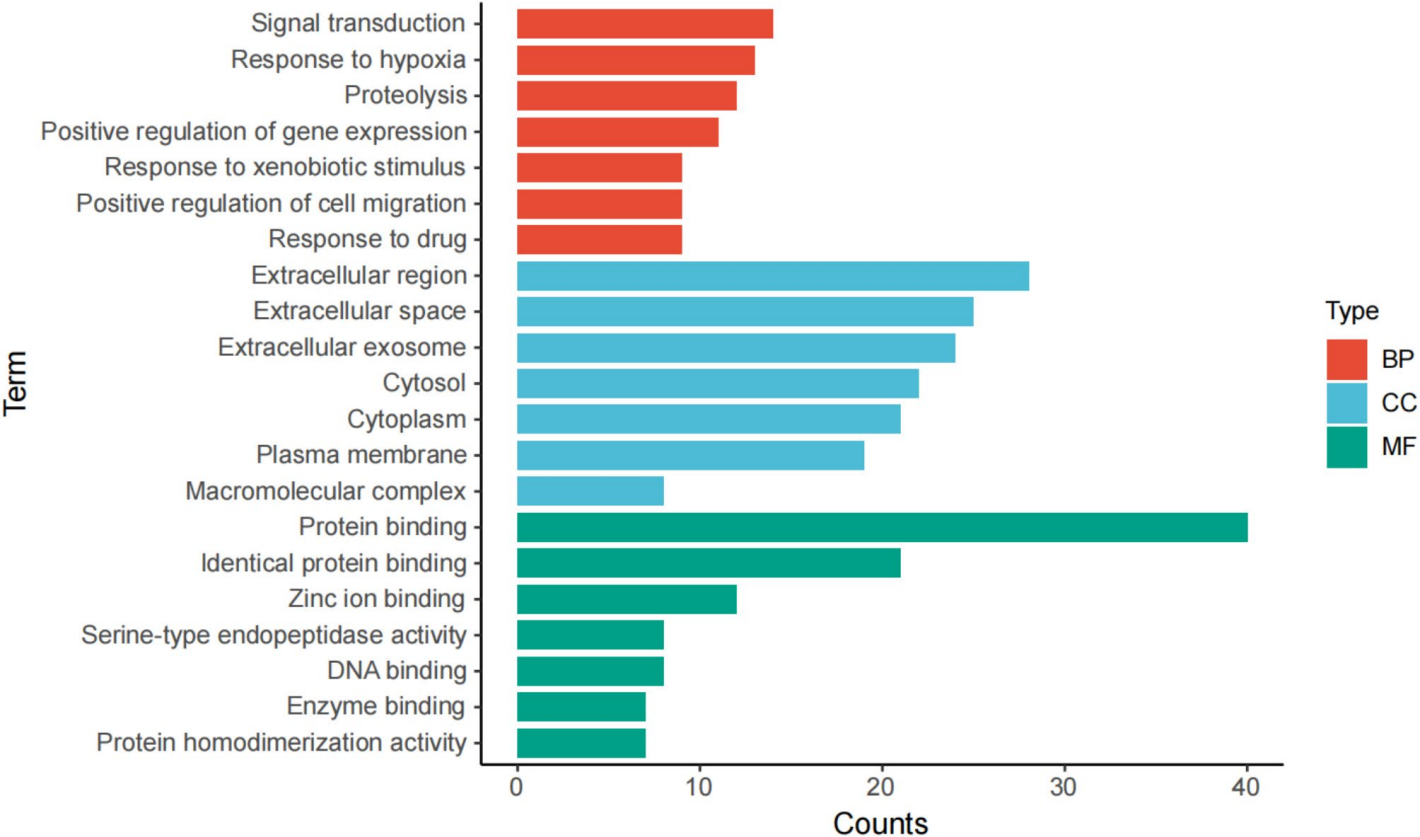
GO functional enrichment analyses of common targets in HCQ-IgAN-albuminuria. BP: biological process; CC: cellular component; MF: molecular function.

**Fig. 5:**
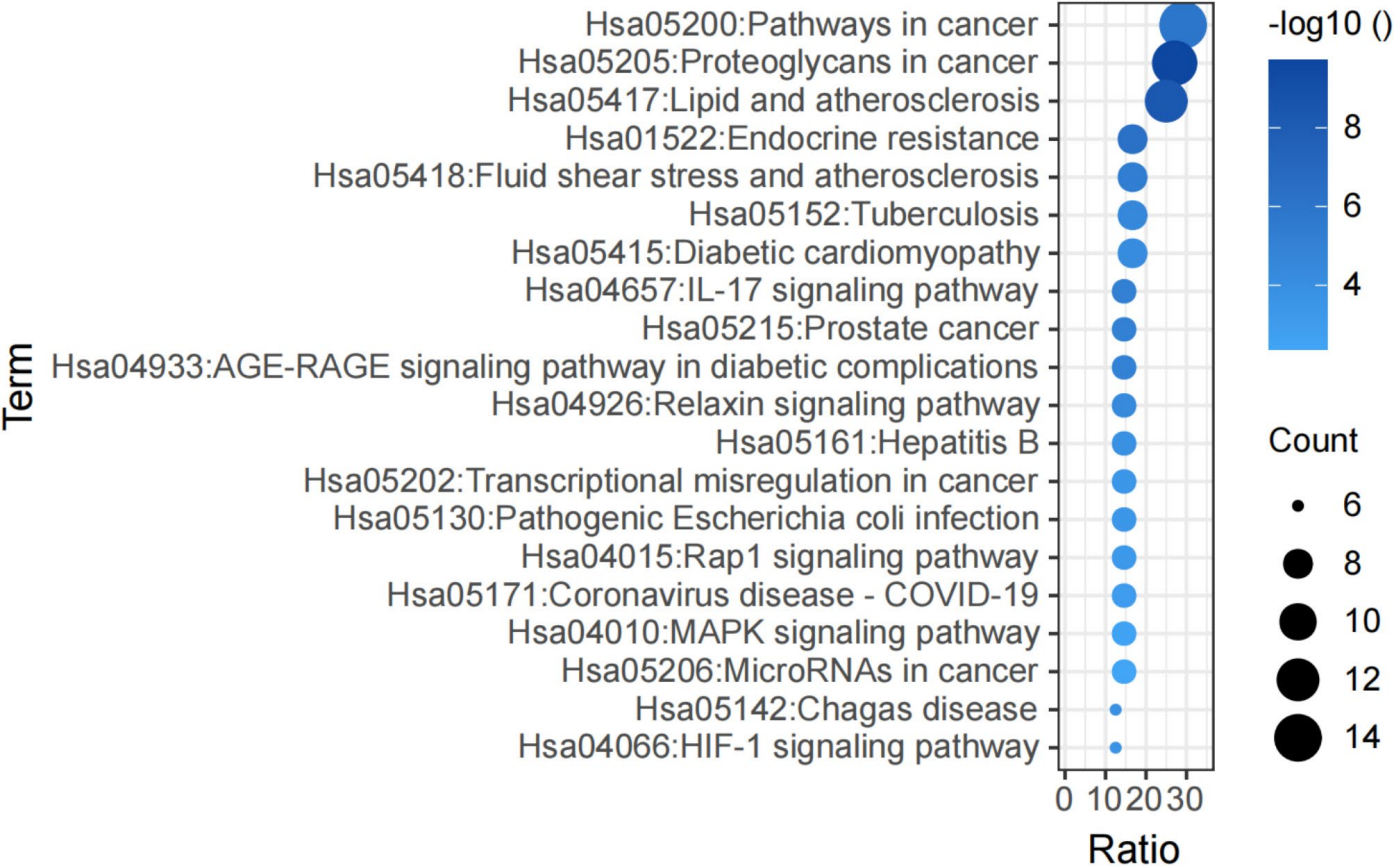
KEGG pathway enrichment analyses of shared targets. Visualization by adjusted *P*-value color scale and genecount dot size.

48 overlapping targets were imported into the DAVID database for subsequent GO and KEGG analyses. With a threshold set at *P* < 0.05, 163 biological processes (BP), 23 cellular components (CC), 46 molecular functions (MF), and 67 signaling pathways were identified. The top 30 GO terms and 20 KEGG terms were selected for further analysis (Figs. 4 and 5). The results indicated that HCQ reduced IgAN proteinuria through involvement in several pathways related to inflammation and oxidative stress, including the AGE-RAGE pathway, IL-17 pathway, and HIF-1 pathway.

### PPI network establishment and analysis

**Fig. 6:**
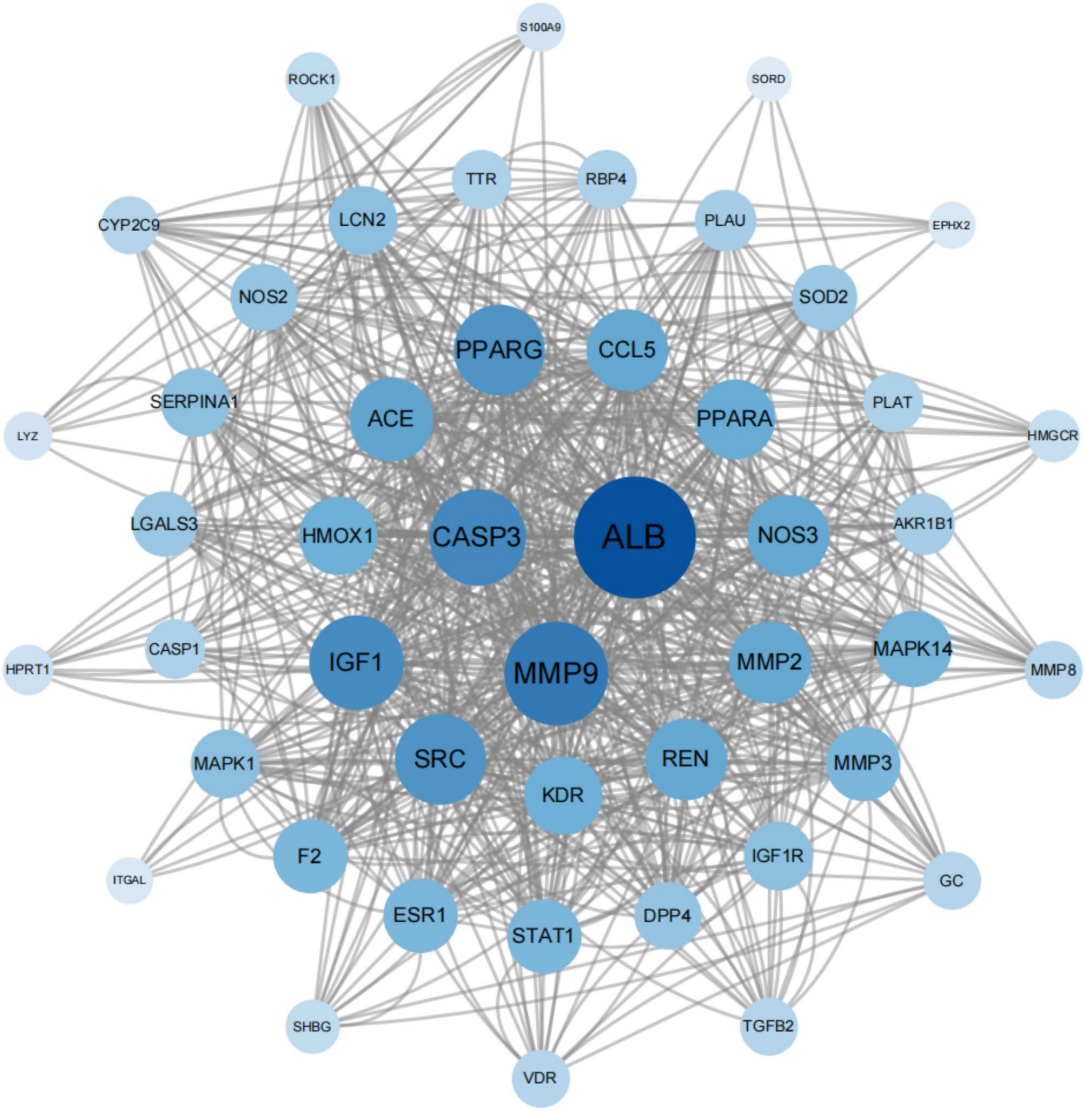
PPI network of 47 shared targets. Nodes represent proteins. Edges represent the intersection between proteins. The colors and node sizes in the network reflected the integrated target number (degree).

To elucidate the protein–protein interaction (PPI) interactions and the underlying mechanisms, a PPI network was constructed from the STRING database and used to analyze the 48 common targets (Fig. 6). Among these, the top 10 targets—ALB, MMP9, CASP3, IGF1, SRC, PPARG, ACE, CCL5, NOS3, and MMP2—were selected for further investigation (Table 2).

**Table 2:** Core targets involved in HCQ-mediated reduction of albuminuria in IgAN.

Target gene	Degree	Betweenness centrality	Closeness centrality
ALB	88	0.23024009989651395	0.9583333333333334
MMP9	68	0.07350751893063553	0.793103448275862
CASP3	60	0.03464736090167956	0.7419354838709677
IGF1	58	0.035812191718549685	0.7301587301587301
SRC	54	0.03031054902863581	0.7076923076923076
PPARG	54	0.03300654728247047	0.7076923076923076
ACE	46	0.02493969340942913	0.6666666666666666
CCL5	44	0.02513925017548206	0.6571428571428571
NOS3	44	0.015134756942088571	0.6571428571428571
MMP2	44	0.011095932106162287	0.647887323943662

### Verification of the GEO dataset

**Fig. 7:**
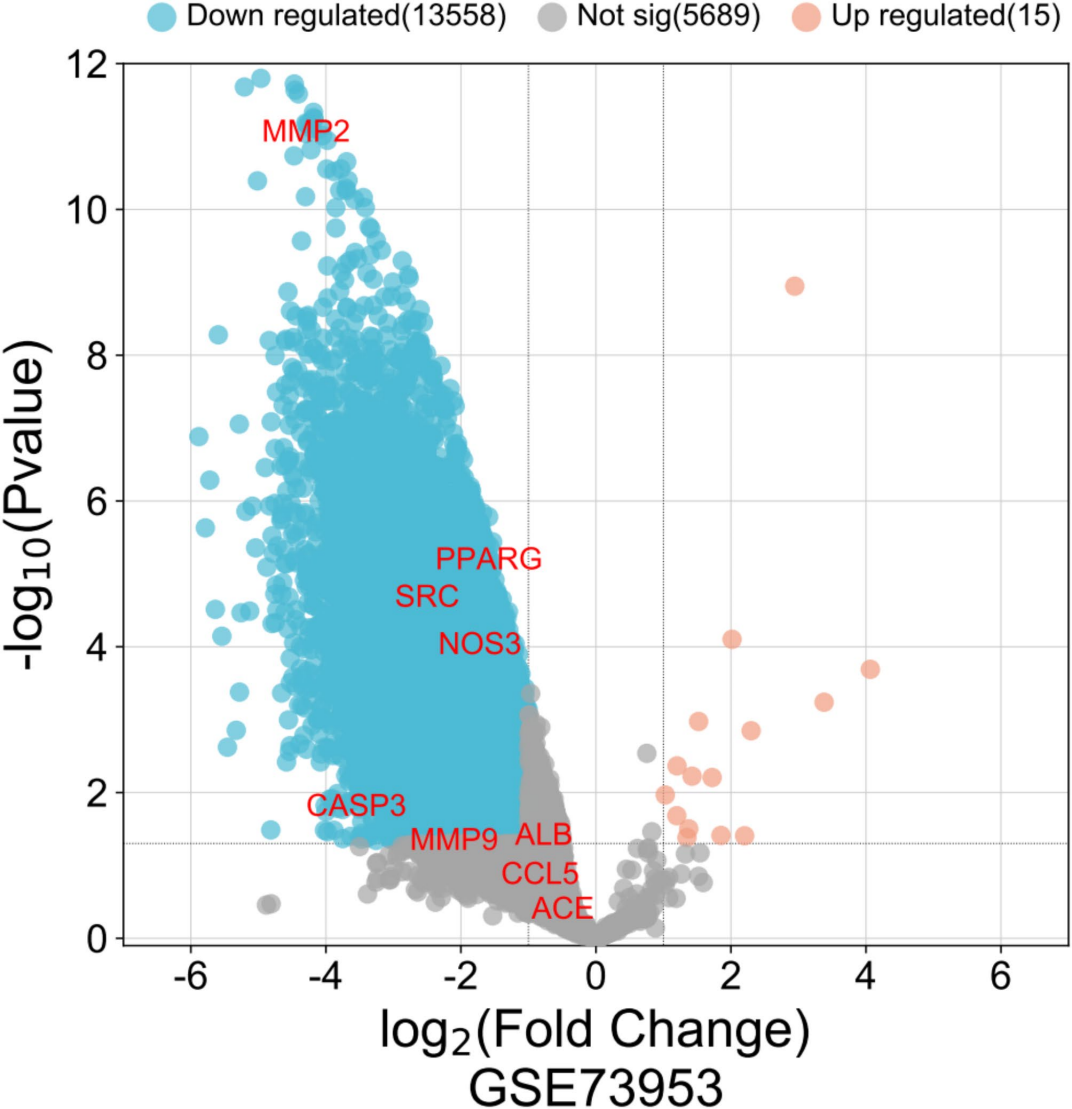
Volcano plot of differentially expressed genes (DEGs) in the GEO dataset GSE73953. Blue represents downregulated DEGs, red represents upregulated DEGs, and grey represents not DEGs.

Differential gene expression analysis was performed on the dataset GSE73953 obtained from the GEO database, comprising 15 samples of IgAN and 16 samples of normal kidney tissues. With *P* < 0.05 and |log2FC| > 2 as the selection criteria, the core genes—CASP3, IGF1, SRC, PPARG, NOS3, and MMP2—were identified as being downregulated in the differential expression analysis (Fig. 7).

### Verification via molecular docking

**Fig. 8:**
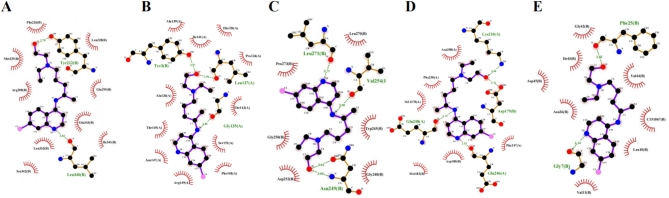
2D molecular docking structures of HCQ with core targets. (**A**): PPARG, (**B**): MMP2, (**C**): NOS3, (**D**): CASP3, (**E**): IGF1. The purple structure represents HCQ.

**Fig. 9:**
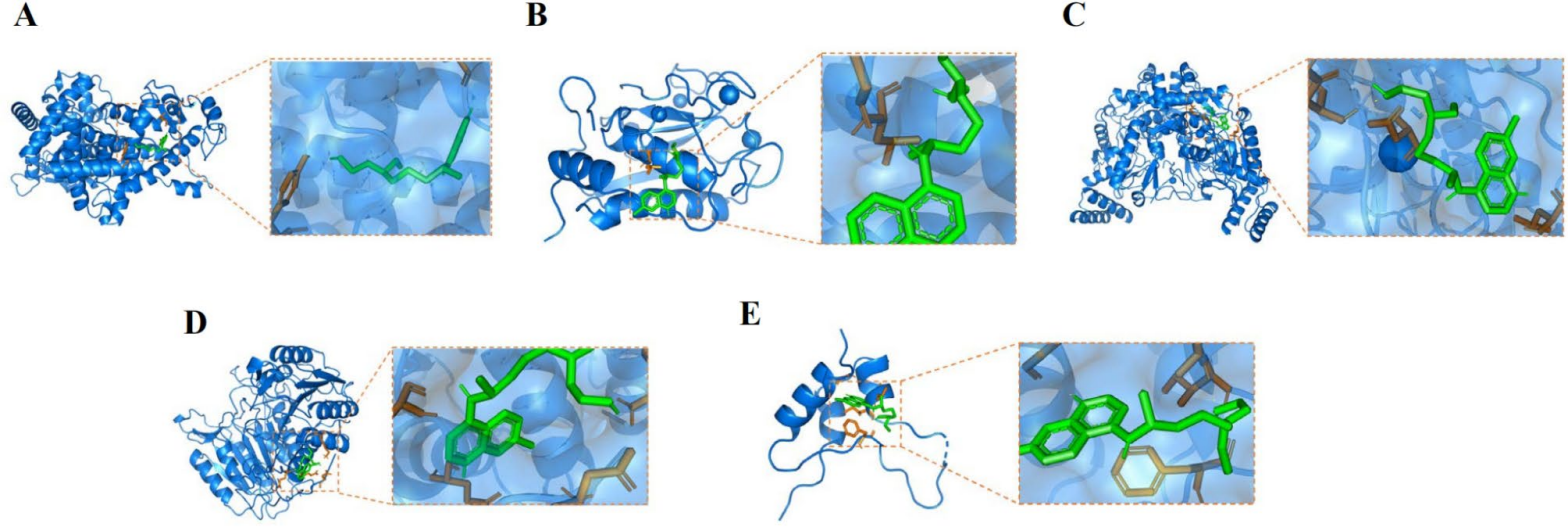
2D molecular docking structures of HCQ with core targets. (**A**): PPARG, (**B**): MMP2, (**C**): NOS3, (**D**): CASP3, (**E**): IGF1. Green represents the chemical structure of HCQ, blue represents the protein, and yellow dashed lines represent the hydrogen bonds formed between HCQ and the protein.

To further verify the results, molecular docking (MD) of the core targets and HCQ was conducted (Table 3). The findings disclosed stable binding structures between HCQ and MMP2, NOS3, PPARG, IGF1, and CASP3, with binding energies below − 5 kcal/mol (Figs. 8 and 9). The interactions of HCQ with these proteins suggested its potential for mitigating albuminuria in individuals with IgAN.

**Table 3:** Molecular docking analysis outcomes.

Target	PDB ID	Binding energy/(kcal/mol)
PPARG	1PRG	− 6.52
MMP2	3AYU	− 6.51
NOS3	3EAH	− 6.02
CASP3	1NMS	− 5.35
IGF1	1GZR	− 5.31
SRC	4U5J	− 4.92

### Construction of the MiRNA networks

**Fig. 10:**
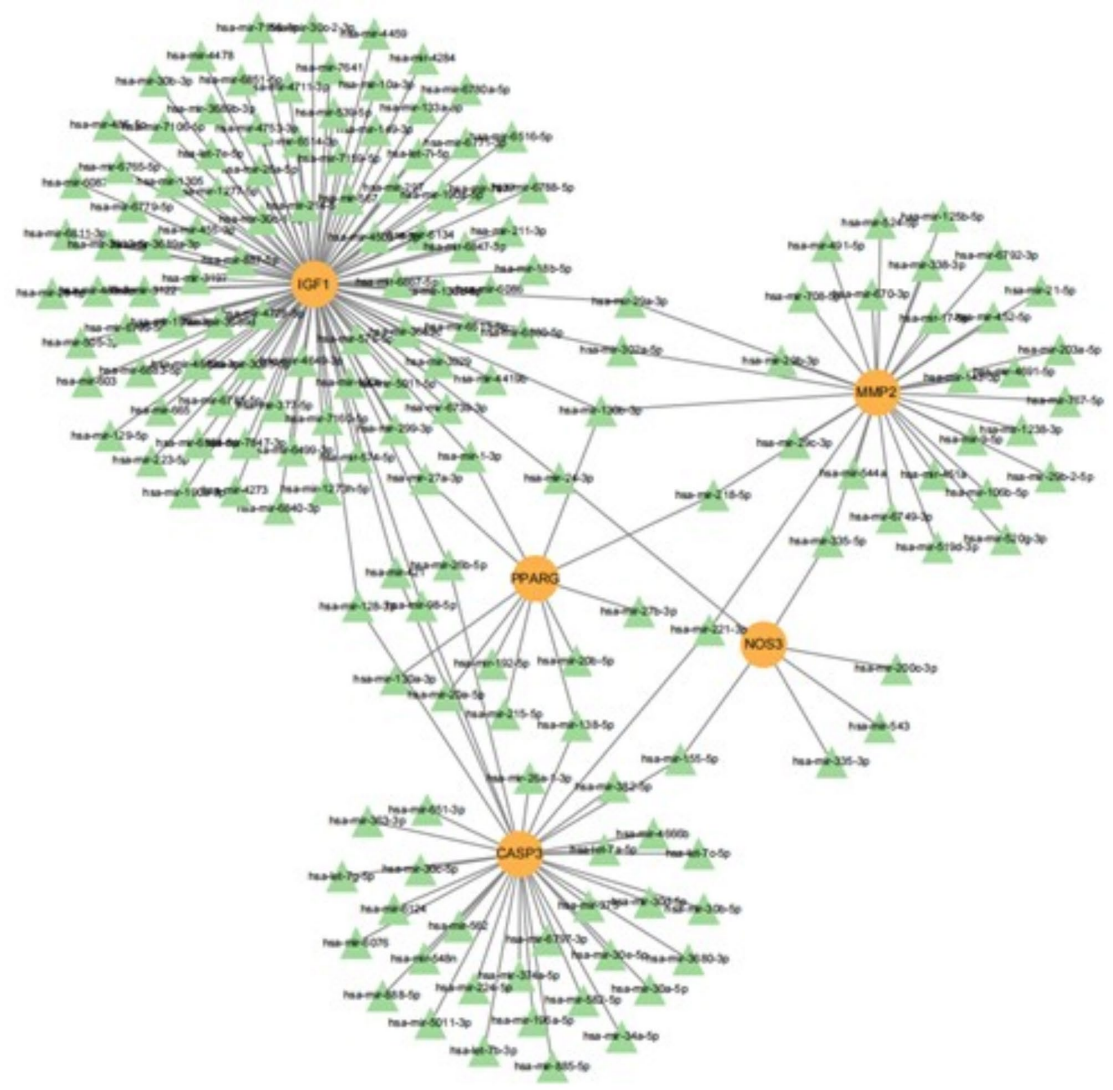
Gene-miRNA network. Orange circular nodes represent genes, and green triangular nodes represent miRNAs.

Gene-miRNA network analysis disclosed a network comprising 171 nodes and 182 interaction lines, encompassing 5 genes and 166 predicted miRNAs. Notably, miR-130b-3p was the sole miRNA found to regulate the expression of more than two genes concurrently, indicating that HCQ may have mitigated albuminuria in IgAN by modulating the expression of MMP2, IGF1, and PPARG via miR-130b-3p (Fig. 10).

## Discussion

Even low-risk IgAN patients were observed to progress to kidney failure within a decade^38^. The KDIGO guidelines highlighted that the management of IgAN predominantly focused on optimizing supportive care; however, this approach may not completely hinder disease progression. Furthermore, they recommended the use of HCQ for Chinese IgAN patients at high risk of progression^39^. While the mechanism by which HCQ reduces proteinuria in IgAN required further exploration to enhance clinical application. Our study employed network pharmacology (NP) and molecular docking (MD) techniques, revealing that HCQ might mitigate proteinuria in IgAN by targeting miR-130b-3p, thereby regulating the expression of MMP2, IGF1, and PPARG.

HCQ is an immunomodulator commonly used to treat autoimmune diseases and is also widely used in lupus nephritis (LN)^40^. The KDIGO guidelines recommended that all systemic lupus erythematosus (SLE) and LN patients should receive HCQ unless there were contraindications^41^. Based on the significant therapeutic effect of HCQ in LN, many researchers turned to IgAN as a treatment target for HCQ in recent years. A randomized controlled study found that the addition of HCQ to RAAS inhibition significantly reduced proteinuria in IgAN patients over a 6-month continuous treatment period^7^. A retrospective study with a 2-year follow-up period found that long-term use of HCQ significantly reduced proteinuria and was safer with fewer side effects compared to systemic corticosteroid therapy^42^. HCQ interfered with the body’s pH value, inhibited the activation of Toll-like receptors (TLRs), and alleviated the inflammatory response by suppressing the TLR/MyD88/NF-κB signaling pathway, thereby diminishing proteinuria in LN^40^. Similarly, as an immune-related disease, the TLR/MyD88/NF-κB signaling pathway also played an important role in the occurrence and development of IgAN^43^.

MiR-130b-3p, located within the human chromosomal region 22q11.22, was originally identified in tumor-related investigations^44^. In the context of kidney diseases, it was determined that miR-130b-3p plays a significant regulatory role in the onset and progression of LN, but there are no studies on miR-130b-3p in IgAN. A study found that miR-130b-3p levels were significantly higher in patients with early-stage LN compared to healthy controls and further elevated in advanced LN. The expression of miR-130b-3p in early LN positively correlated with the renal chronicity index and 24-h proteinuria levels. Additionally, transfection of miR-130b-3p inhibitor increased E-cadherin expression, decreased α-SMA expression, inhibited epithelial-mesenchymal transition, and ameliorated renal fibrosis^45^. Previous studies demonstrated that the activation of the TLR/MyD88/NF-κB signaling pathway was intimately associated with the development of renal fibrosis, and that renal fibrosis and damage in the LN mouse model could be mitigated by inhibiting the TLR9/MyD88/NF-κB signaling pathway^46^. Synthesizing these findings with prior research, the TLR/MyD88/NF-κB signaling pathway was identified as pivotal in the pathogenesis of IgAN. Our study discovered that miR-130b-3p played a crucial role in IgAN. Consequently, we hypothesized that miR-130b-3p might be involved in the pathogenesis and progression of IgAN by modulating the TLR/MyD88/NF-κB signaling pathway.

Our research revealed that HCQ might mitigate proteinuria in IgAN by modulating the expression of MMP2, IGF1, and PPARG via miR-130b-3p. Additionally, the TLR/MyD88/NF-κB signaling pathway was identified to influence the expression of MMP2, IGF1, and PPARG, thereby regulating cell proliferation and fibrosis in IgAN. In IgAN, MMP2 participated in the damage and repair processes of the glomerulus and tubulointerstitium, encompassing the degradation of extracellular matrix (ECM) and the synthesis of new matrix, which impacted the proliferation and fibrosis of renal cells^47^. Previous studies had demonstrated that miR-130b-3p could modulate the expression of MMP2, thereby regulating the proliferation, migration, and invasion of bladder cancer cells^48^. IGF-1 was shown to influence the infiltration of renal interstitial inflammatory cells and the accumulation of fibroblasts, contributing to the development of renal interstitial fibrosis^49^. MiR-130b-3p was found to regulate the secretion of IGF-1, thus affecting fibroblast activity^50^. The activation of PPARG was suggested to protect renal function by enhancing energy metabolism and reducing oxidative stress in IgAN^51^, and several studies had identified that miR-130b-3p could directly target and modulate PPARG expression^52^. Synthesizing the literature with our study findings, we hypothesized that miR-130b-3p impacts the TLR/MyD88/NF-κB signaling pathway, thereby regulating the expression of MMP2, IGF1, and PPARG.

In conclusion, our study revealed that miR-130b-3p was a key intersecting miRNA in the pharmacological mechanism of HCQ and the pathogenesis of IgAN. Integrating our findings with prior discussions, we hypothesized that HCQ might exert therapeutic effects on IgAN by modulating the levels of miR-130b-3p. Synthesizing our results with previous studies, we postulated that HCQ targeted miR-130b-3p, thereby influencing the TLR/MyD88/NF-κB signaling pathway and regulating the expression of MMP2, IGF1, and PPARG, which could contribute to the reduction of IgAN proteinuria.

Nevertheless, our study had limitations, as cell and animal experiments were not conducted due to budgetary constraints. Further experimental validation was necessary to ascertain whether HCQ modulated the expression of MMP2, IGF1, and PPARG genes via miR-130b-3p, thereby influencing the TLR4/NF-κB/MAPK signaling pathway and potentially mitigating albuminuria in IgAN. Moreover, the potential of miR-130b-3p as a novel non-invasive biomarker for assessing disease progression and prognosis needed to be validated.

## Conclusion

HCQ targeted miR-130b-3p, thereby influencing the TLR/MyD88/NF-κB signaling pathway and regulating the expression of MMP2, IGF1, and PPARG, which contributed to the reduction of IgAN proteinuria.

## Data Availability

All data generated or analysed during this study are included in this article.
